# Emerging roles of RNA N^4^-acetylcytidine modification in reproductive health

**DOI:** 10.1093/procel/pwaf013

**Published:** 2025-02-19

**Authors:** Zibaguli Wubulikasimu, Hongyu Zhao, Fengbiao Mao, Xiaolu Zhao

**Affiliations:** State Key Laboratory of Female Fertility Promotion, Center for Reproductive Medicine, Department of Obstetrics and Gynecology, Peking University Third Hospital, Beijing 100191, China; National Clinical Research Center for Obstetrics and Gynecology, Peking University Third Hospital, Beijing 100191, China; Key Laboratory of Assisted Reproduction (Peking University), Ministry of Education, Beijing 100191, China; Beijing Key Laboratory of Reproductive Endocrinology and Assisted Reproductive Technology, Beijing 100191, China; State Key Laboratory of Female Fertility Promotion, Center for Reproductive Medicine, Department of Obstetrics and Gynecology, Peking University Third Hospital, Beijing 100191, China; National Clinical Research Center for Obstetrics and Gynecology, Peking University Third Hospital, Beijing 100191, China; Key Laboratory of Assisted Reproduction (Peking University), Ministry of Education, Beijing 100191, China; Beijing Key Laboratory of Reproductive Endocrinology and Assisted Reproductive Technology, Beijing 100191, China; Institute of Medical Innovation and Research, Peking University Third Hospital, Beijing 100191, China; Cancer Center, Peking University Third Hospital, Beijing 100191, China; Beijing Key Laboratory for Interdisciplinary Research in Gastrointestinal Oncology (BLGO), Beijing 100191, China; State Key Laboratory of Female Fertility Promotion, Center for Reproductive Medicine, Department of Obstetrics and Gynecology, Peking University Third Hospital, Beijing 100191, China; National Clinical Research Center for Obstetrics and Gynecology, Peking University Third Hospital, Beijing 100191, China; Key Laboratory of Assisted Reproduction (Peking University), Ministry of Education, Beijing 100191, China; Beijing Key Laboratory of Reproductive Endocrinology and Assisted Reproductive Technology, Beijing 100191, China

**Keywords:** epigenetic regulation, RNA modification, ac^4^C, gynecological disease, reproduction

## Abstract

N^4^-acetylcytidine (ac^4^C), an emerging posttranscriptional RNA modification, plays a pivotal role in epigenetic regulation. Ac^4^C is detected not only in tRNA, rRNA, and mRNA, but also in miRNA, lncRNA, viral RNA, and even DNA. Functionally, ac^4^C stabilizes mRNA, enhances protein translation fidelity, and impacts various biological processes and diseases such as cancer, inflammation, immune regulation, neural diseases, osteogenic differentiation, cardiovascular diseases, viral infections, and replication. Current research primarily focuses on ac^4^C’s roles in cancer progression and immunity, with emerging findings in gynecological diseases and reproduction. However, a comprehensive understanding of ac4C’s implications in reproductive health is lacking. This review provides a historical perspective on ac^4^C’s discovery and detection methods, elucidates its functions in reproductive development and gynecological disorders, and offers insights for further research in reproductive health. This review aims to pave the way for innovative therapeutic approaches and precise diagnostic tools tailored to this field.

## Introduction

Posttranscriptional RNA modifications are essential components of epigenetic regulation, playing diverse roles in various biological processes. To date, more than 170 chemical modifications of RNA have been identified, including methylation, acetylation, deamination, isomerization, and oxidation, spanning across different RNA types in mammals, bacteria, and fungi (Cui et al., 2022; Li et al., 2023; Tsai and Cullen, 2020). These modifications orchestrate dynamic changes facilitated by two classes of enzymes known as “writers” and “erasers,” ultimately exerting their functions through RNA-binding proteins (RBPs) referred to as “reader” proteins (Cui et al., 2022; Wang et al., 2023a). Early investigations into RNA modifications primarily centered on ribosomal RNA (rRNA) and transfer RNA (tRNA) due to technical constraints in detecting mRNA modifications. These modifications often occur at highly conserved sites and can influence ribosome maturation and tRNA stability (Ito et al., 2014a; Oashi et al., 1972; Stern and Schulman, 1978; Zachau et al., 1966). With the advancement of sequencing technologies in recent years, a growing number of mRNA modifications have been unveiled. Common eukaryotic mRNA modifications, such as N6-methyladenosine (m^6^A), N^4^-acetylcytidine (ac^4^C), pseudouridine (Ψ), inosine (I), 5-hydroxymethylcytidine (hm^5^C), 7-methylguanosine (m^7^G), 2′-*O*-methylnucleoside (Nm), uridylation, and adenosine-to-inosine (A-to-I) RNA editing, intricately regulate key RNA metabolic processes like stability, translation, and alternative splicing, thereby exerting precise control over gene expression and impacting a wide array of cellular and biological functions (Li et al., 2023; Sun et al., 2023; Thalalla Gamage et al., 2024; Tsai and Cullen, 2020; Wiener and Schwartz, 2020). Among these modifications, m^6^A stands out as the most prevalent internal posttranscriptional modification (PTM) found in mRNA across various organisms. Extensively studied, m^6^A not only significantly influences RNA fate and metabolism but also plays a broader role in epigenetic regulation, formation of genomic structure, and maintenance of genomic stability. Disruption of m^6^A has been linked to various disorders affecting processes such as embryo development, cell fate determination, innate immune responses, and neurobehavioral functions (An and Duan, 2022; Zaccara et al., 2019). In contrast to the well-explored landscape of m^6^A modification, ac^4^C modification represents a burgeoning area in epitranscriptomic mark that is still in its early stages of exploration.

**Figure 1.**
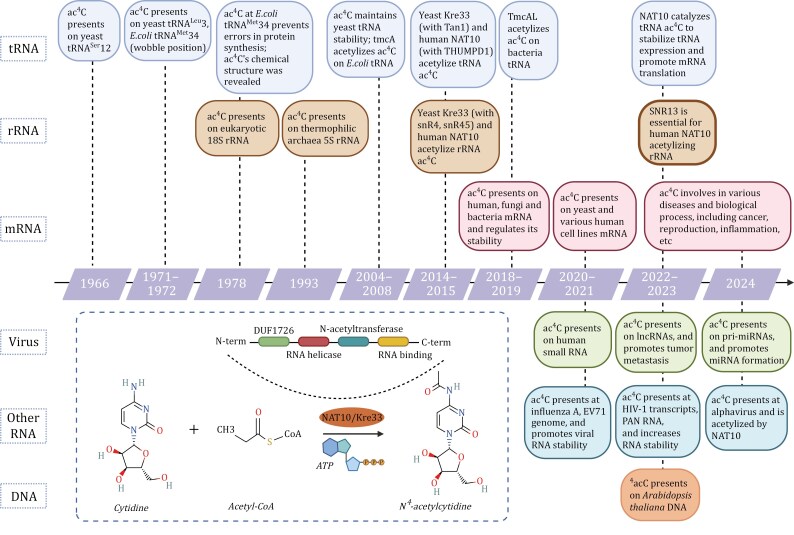
**The discovery history of ac** ^**4**^ **C**. Notable studies on ac^4^C are listed with a timeline and color-coded based on the nucleoside type in which ac^4^C is present. The conversion from 4C to ac^4^C is depicted in the frame (created by BioRender.com and PubChem).

Ac^4^C, the sole identified acetylation modification in eukaryotic RNA, has been found to be highly conserved across eukaryotes and prokaryotes. Initially detected in tRNA and 18S rRNA around the 1970s, ac^4^C has since been identified in yeast, bacteria and human, ensuring precise protein translation (Igo-Kemenes and Zachau, 1969; Kowalski et al., 1971; Stern and Schulman, 1978; Thomas et al., 1978; Zachau et al., 1966). Recent studies have revealed abundant ac^4^C in human poly(A) RNA, enhancing mRNA stability and translation efficiency (Arango et al., 2018). The *N*-acetyltransferase 10 (NAT10; yeast homolog: Kre33), possessing acetyltransferase activity and RNA-binding capabilities (Fig. 1), serves as the primary ac^4^C writer enzymes for rRNA, tRNA, and mRNA. Additionally, THUMPD1 and SNORD13 have been identified as essential cofactors for ac^4^C formation on tRNA and rRNA, respectively (Bortolin-Cavaillé et al., 2022; Sharma et al., 2015, 2017).

The NAT10-mediated ac^4^C modification in mRNA plays crucial roles in the progression of various human diseases. Fluctuations in ac^4^C levels have been linked to inflammatory, autoimmune, neural diseases, cardiovascular diseases, and metabolic conditions, showcasing its potential as a biomarker for disease states. Moreover, ac^4^C modifications mediated by NAT10 have been implicated in the initiation and advancement of diverse cancers by influencing mRNA stability, translation efficiency, and DNA repair processes, potentially unveiling new mechanisms and therapeutic targets for cancer therapy (Boo and Kim, 2020; Cui et al., 2022; Jin et al., 2020; Liu et al., 2024b; Wang et al., 2023a). Recent studies have underscored the significant involvement of ac^4^C in shaping reproductive health, with elevated ac^4^C levels observed in patients with various reproductive disorders (Szymańska et al., 2010; Zhang et al., 2012). The role of NAT10-mediated ac^4^C in regulating oocyte maturation, embryonic stem cell pluripotency, and spermatogonial cell development into meiosis has also been highlighted (Chen et al., 2022; Jiang et al., 2023; Xiang et al., 2021).

While existing reviews have summarized the role of ac^4^C in conditions like cancer and inflammation, a comprehensive review focusing on its functional implications in reproductive development is currently lacking (Boo and Kim, 2020; Zhang et al., 2024c). In this review, we aim to provide a detailed overview of the historical discovery and detection methodologies of ac^4^C, shedding light on its functions in reproductive health and gynecological disorders. By elucidating the roles of ac^4^C in these contexts, we aim to pave the way for the development of novel therapeutic strategies and diagnostic tools tailored to reproductive health.

## Discovery history of ac^4^C modification

**Figure 2.**
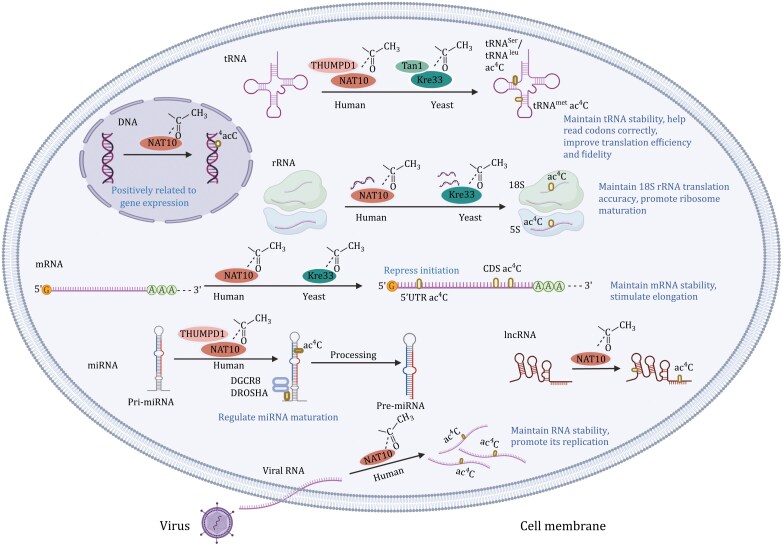
**The presence of ac** ^**4**^ **C on various nucleic acids.** The writer of ac^4^C on various nucleosides (including tRNA, rRNA, mRNA, miRNA, lncRNA, viral genome, and DNA), and the main roles of ac^4^C, are illustrated, respectively (created by BioRender.com).

Ac^4^C is recognized as the principal acetylation modification in posttranscriptional processes, representing a pivotal advancement in scientific inquiry (Fig. 1). Initially, investigations into ac^4^C modification primarily concentrated on bacterial and fungal tRNA and rRNA. The identification of NAT10 extended the exploration of ac^4^C modification from prokaryotic contexts to mammalian RNA systems (Fig. 2). Here, we will delineate the timeline of ac^4^C research across diverse nucleic acid categories.

### Transfer RNA

The identification of ac^4^C modification on tRNA stands as a significant milestone in the realm of RNA epigenetics. In 1966, Zachau and colleagues discovered two variants of serine transfer RNA (tRNA^Ser^) and unveiled the pioneering presence of ac^4^C at position 12 in brewer’s yeast tRNA^Ser^ (Zachau et al., 1966). Subsequent studies revealed the specific reactivity of ac^4^C on tRNA^Ser^ with sodium borohydride (Igo-Kemenes and Zachau, 1969). Kowalski and collaborators later identified ac^4^C on yeast tRNA through the analysis of the nucleotide sequence of “denaturable” leucine acceptor tRNA^Leu3^ from baker’s yeast (Kowalski et al., 1971). Oashi and coworkers pinpointed ac^4^C at the wobble position of *Escherichia coli* tRNA^Met^ (Oashi et al., 1972). Following these discoveries, research on ac^4^C modification in tRNA has continued to evolve. Stern and Schulman elucidated the positioning of the ac^4^C modification on *E*. *coli* tRNA at the wobble position of the initiator tRNA^Met^, thereby enhancing translation fidelity by favoring pairing with the AUG codon for methionine over the AUA codon for isoleucine (Stern and Schulman, 1978). This functional role of ac^4^C was associated with its molecular conformation, as further elucidated by Kwai and colleagues in 1989 (Kawai et al., 1989). Their characterization of the molecular conformation of ac^4^C using three-dimensional X-ray techniques revealed a pseudo-bicyclic conformation, influencing Watson–Crick base pairing and mismatch discrimination (Parthasarathy et al., 1978). The presence of ac^4^C at the wobble position of *E*. *coli* tRNA^Met^ stabilizes the ribose C3′ endomorphism, strengthening C-G codon–anticodon base pairing to uphold the tertiary structure of tRNA and ensure accurate codon reading. Johansson and Byström delved into the process of ac^4^C formation in yeast, identifying the essential role of the *Tan1* gene in the synthesis of ac^4^C in tRNA^Leu^ and tRNA^Ser^ (Johansson and Byström, 2004).

Mutations affecting ac^4^C at tRNA^Ser^12 in *Saccharomyces cerevisiae* have been linked to reduced levels of mature tRNA^Ser^. Additionally, the simultaneous disruption of ac^4^C and another tRNA modification, m^7^G, led to tRNA^Ser^ instability in mutant yeast strains, hindering strain growth (Kotelawala et al., 2008). The above observation underscores the crucial role of ac^4^C in maintaining yeast tRNA stability. Ikeuchi and coauthors initially discovered tmcA (tRNA^Met^ cytidine acetyltransferase), encoded by the *ypfI* gene and comprises the GCN5-associated acetyltransferase (GNAT) domain and the walker-type ATPase domain, as the enzyme responsible for catalyzing the formation of ac^4^C in bacterial swing-base tRNA^Met^ in the presence of acetyl-CoA (Ikeuchi et al., 2008). Moreover, the ATPase domain, which is part of the RNA helicase module fused to the GNAT domain, potentially unwinds the stem-loop structure of tRNA to aid in GNAT domain-mediated ac^4^C formation (Ikeuchi et al., 2008; Taniguchi et al., 2018).

Homologs of TmcA are widely distributed in archaea and eukaryotes. Sharma and collaborators identified Kre33 (encoded by the *Rra1p* gene) and NAT10 (encoded by the *NAT10* gene) as homologs of TmcA, functioning as acetyltransferases responsible for catalyzing ac^4^C formation on tRNA in yeast and mammals, respectively (Ikeuchi et al., 2008; Sharma et al., 2015). These enzymes belong to the GNAT-type enzyme family and consist of approximately 1,000 amino acid proteins containing a helicase domain, a GNAT domain, and multiple predicted RNA-binding domains (Fig. 1) (Thomas et al., 2019). Notably, their activity is reliant on specific conserved cofactors. The yeast Tan1 assists Kre33 in ac^4^C synthesis on yeast tRNA^Leu^ and tRNA^Ser^ but does not participate in rRNA acetylation. Human THUMPD1 is essential for ac^4^C formation on tRNA catalyzed by NAT10 (Johansson and Byström, 2004; Sharma et al., 2015).

Recently, TmcAL, an enzyme encoded by the *ylbM* gene in bacteria, has been identified as a novel RNA acetylase responsible for catalyzing ac^4^C formation at the tRNA^Met^ wobble site (position 34) in *Bacillus subtilis* (Taniguchi et al., 2018). Unlike TmcA, TmcAL lacks the helicase domain, GNAT domain, or the cofactor acetyl-CoA. Its catalytic mechanism resembles that of aminoacyl-tRNA synthases, activating acetic acid ions to form acetyl groups and transferring them to tRNA to generate ac^4^C34 (Taniguchi et al., 2018). Building upon previous research by Stern and Schulman in 1978, which highlighted the role of ac^4^C at the wobble position of tRNA^Met^ in preventing misreading of the AUA codon, Taniguchi and colleagues discovered that ac^4^C, in conjunction with the L34 tRNA^Ile^, collaborates to regulate the decoding ability and efficiency of tRNA, ensuring high fidelity during protein synthesis (Stern and Schulman, 1978; Taniguchi et al., 2018).

Wei and colleagues reported that the deletion of NAT10 resulted in reduced ac^4^C expression and decreased levels of most ac^4^C-modified tRNAs, leading to a diminished rate of new protein synthesis (Wei et al., 2023). This highlights the role of NAT10 in catalyzing tRNA ac^4^C modification to stabilize tRNA expression and enhance mRNA translation. The above functional impact spans across different organisms, from fungi to archaea. In a hyperthermophilic archaeon (*T*. *kodakarensis*), mutations affecting ac^4^C in tRNA have been linked to a decrease in cellular hyperthermal tolerance (Orita et al., 2019). Notably, the levels of ac^4^C in *T*. *kodakarensis* exhibited a significant increase with rising temperatures, and strains lacking the acetyltransferase NAT10 displayed temperature-dependent growth impairments (Sas-Chen et al., 2020).

### Ribosomal RNA

Thomas and colleagues were the first to identify ac^4^C modification in the small subunit of rat 18S rRNA using two-dimensional thin-layer chromatography (Thomas et al., 1978). Subsequent studies confirmed the presence of ac^4^C in bacterial 5S rRNA and yeast 18S rRNA through liquid chromatography-mass spectrometry (LC-MS) (Bruenger et al., 1993; Ito et al., 2014a). Further investigations by Sharma and collaborators unveiled the existence of ac^4^C modifications on the 18S rRNA of human HEK293 cells, human HCT116 cells, and yeast. Additionally, they identified two acetylated cytosine residues in helix 34 and helix 45 of 18S rRNA. Helix 34 was deemed crucial for translation accuracy, while helix 45 was positioned near the decoding site, both playing significant roles in decoding 18S rRNA and preserving translation fidelity (Ito et al., 2014a; Sharma et al., 2015).

Similar to tRNA, the ac^4^C modification on rRNA is catalyzed by yeast Kre33 and mammalian NAT10. In *S. cerevisiae*, Kre33 facilitates the formation of ac^4^C at position 1,773 in 18S rRNA, while NAT10 catalyzes the ac^4^C modification at position 1,842 in human HEK293 cells (Ito et al., 2014a, 2014b; Sharma et al., 2015; Taoka et al., 2014). Unlike tRNA, the process of ac^4^C formation on rRNA requires small nucleolar RNAs (snoRNAs) rather than Tan1/THUMPD1. SnoRNAs are a class of noncoding RNA molecules ranging from 60 to 1,000 nm in length, serving as scaffolds for assembling conserved core proteins. They are categorized into box C/D, box H/ACA, and MRP based on evolutionarily conserved sequence elements. Notably, two orphan box C/D snoRNAs, snR4 and snR45, specifically guide Kre33 to ac^4^C targets in yeast rRNA for cytosine acetylation, with snR4 targeting ac^4^C1280 and snR45 targeting ac^4^C1773 (Sharma et al., 2015, 2017).

In humans, the vertebrate-specific box C/D snoRNA U13 (SNORD13) serves as the homolog of snR45. Sharma et al. confirmed the essential role of SNORD13 in human cells for the acetylation of a single cytidine residue (ac^4^C1842) in helix 45 of the small-subunit rRNA (Bortolin-Cavaillé et al., 2022). These snoRNAs, including snR45, snR4, and SNORD13, base-pair with the 18S rRNA cytidine through two imperfect antisense elements, facilitating the exposure of the substrate cytosine for acetylation by NAT10/Kre33 at this site through an unknown mechanism. This process indirectly contributes to rRNA acetylation and pre-rRNA folding (Bortolin-Cavaillé et al., 2022; Sharma et al., 2015, 2017). Although the ac^4^C formation in human 18S rRNA helix 45 requires the coordinated activity of NAT10 and SNORD13, there is no direct evidence of their interaction. Furthermore, SNORD13 has been utilized to modulate RNA-guided cytidine acetylation. Gamage et al. drove the acetylation of ectopic pre-rRNA substrates that were not effectively modified in endogenous cells by introducing an SNORD13 mutant (Thalalla Gamage et al., 2022). Mutations in NAT10 in yeast strains result in a slow-growth phenotype and a deficiency in the maturation of small-subunit rRNA from precursor RNA, indicating the essential role of cytidine acetylation in yeast ribosome assembly (Taoka et al., 2014). Conversely, Sharma et al. demonstrated that SNORD13-dependent ac^4^C modification is dispensable for human cell growth, ribosome biogenesis, translation, and development (Bortolin-Cavaillé et al., 2022; Sharma et al., 2015). Therefore, further investigations are warranted to elucidate the biological functions and processes of ac^4^C modification in rRNA across different species.

### Messenger RNA

Arango et al. (2018) were the first to identify ac^4^C in the human HeLa cell transcriptome at over 4,000 sites using acRIP-seq. Their findings revealed that ac^4^C was predominantly concentrated at the 5′ end of the coding sequence (CDS) with some occurrences near the 3′ end of the untranslated region (UTR). Notably, a biased representation of cytidine within wobble sites that was empirically determined to influence mRNA decoding efficiency. Their research suggested that ac^4^C plays a crucial role in extending mRNA half-life, enhancing its stability, and ultimately facilitating translation. Subsequent studies by Arango et al. further indicated that the impact of ac^4^C on mRNA translation is position-dependent (Arango et al., 2022). Specifically, ac^4^C within the CDS region of mRNA was found to enhance stability and promote translation. Conversely, ac^4^C in the 5′UTR region could impede translation initiation by augmenting upstream translation initiation sites (upTIS) and repressing annotated TIS (aTIS), such as the canonical initiation codon AUG, thereby influencing mRNA interactions with tRNA/ribosomes. Furthermore, ac^4^C within Kozak sequences could directly influence tRNA_i_^Met^ interactions by forming inhibitory structures, leading to significant translation inhibition *in vitro*.

As research in this area expands rapidly, the presence of ac^4^C on mRNA has been observed in various mammalian cell lines, including cancer cells, immune cells, and germ cells, as well as in fungi and bacteria (Guo et al., 2020; Jin et al., 2020; Xiang et al., 2021). The presence of ac^4^C modification has been shown to be conserved throughout all organisms (Tardu et al., 2019). In mammals, NAT10 serves as the sole known acetyltransferase responsible for ac^4^C modification, while in yeast, the NAT10 homologous protein Kre33 (Rra1) is involved in its generation. Additionally, mRNA ac^4^C levels in yeast exhibited dynamic changes in response to conditions like heat shock, glucose deprivation, or oxidative stress (Arango et al., 2018; Tardu et al., 2019).

### Other RNA

Lan et al. (2018) utilized LC-ESI-MS/MS to identify 24 nucleotide modifications in small RNAs derived from human cells, among which ac^4^C was identified for the first time in small RNAs. They revealed that ac^4^C modification on miRNA is catalyzed by NAT10/THUMPD1, playing a crucial role in the biological production process of miRNA. Mature miRNAs originate from longer primary transcripts that undergo cleavage and processing by a series of nucleases. The presence of ac^4^C on pri-miRNA enhances the interaction between pri-miRNA and DGCR8, facilitating the conversion of pri-miRNA into precursor miRNA (pre-miRNA) and thereby enhancing the biogenesis of mature miRNA (Zhang et al., 2024b).

Furthermore, beyond its impact on short RNAs, NAT10-mediated ac^4^C modification also occurred on the long noncoding RNAs (lncRNAs), enhancing the stability and expression of lncRNAs (Yu et al., 2023). These findings underscored the critical role of ac^4^C modification in regulating the stability and expression of both short and long noncoding RNAs, highlighting its significance in RNA processing and function.

### Virus

Multiple chemical modifications are present in RNA virus genomes, playing essential roles in RNA function and metabolism. PTM pathways are crucial in the host response to viral infections, influencing the virus’s infection cycle and regulating the antiviral innate immune process. This can be regarded as a novel regulatory system for RNA viruses to invade the host (Li and Rana, 2022; Shen and Zhang, 2023).

The first ac^4^C residues identified on a virus genome were found in the Human immunodeficiency virus type 1 (HIV-1). HIV-1 replication relies on NAT10-associated Tat, a virus-encoded regulatory protein that activates virus transcription (Jean et al., 2017). Tsai et al. demonstrated that HIV-1 transcripts harbor ac^4^C residues at multiple sites and utilize host NAT10 to add ac^4^C to viral RNA (Tsai et al., 2020). Deletion or mutation of NAT10-mediated ac^4^C resulted in decreasing of RNA stability and replication level of HIV-1, indicating that NAT10-mediated ac^4^C enhances HIV-1 RNA replication by increasing its stability. Enterovirus 71 (EV71) genome also underwent a NAT10-mediated ac^4^C modification in its 5′UTR (Hao et al., 2022). Inhibition of NAT10 or ac^4^C sites on internal ribosomal entry sites (IRES) suppressed EV71 replication and reduced the pathogenicity of ac^4^C-deficient mutant EV71 *in vivo*. Mechanistically, ac^4^C promoted viral RNA translation and enhanced RNA stability by selectively recruiting PCBP2 to IRES and increasing RNA-dependent RNA polymerase binding to viral RNA.

Furuse et al. mapped RNA modifications in A549 cells infected with influenza A virus using RNA immunoprecipitation combined deep sequencing methods (Furuse, 2021). They identified potential regions for ac^4^C in the negative strand segments of viral genomic RNA, and observed the enrichment of ac^4^C at the 5′UTR of the host’s DAZAP1 gene. They also discovered the reducing of the host factor NAT10 in human cells infected with the influenza A virus negatively regulated RNA stability and viral growth by interacting with viral proteins, PB1, NP, NA, and M1 and regulate ac^4^C. Dang et al. investigated the regulation of viral replication via RNA acetylation in host mRNA (Dang et al., 2024). They found that NAT10 and ac^4^C levels are promoted in cells following alphavirus infection, while the deletion or inhibition of NAT10 reduced the replication of alphavirus. NAT10 enhances alphavirus replication by maintaining the stability of host lymphocyte antigen 6 family member E mRNA, a multifunctional interferon-stimulating gene that promotes alphavirus replication, showcasing an unconventional role of ac^4^C modification in regulating host mRNA stability rather than viral mRNA.

Apart from RNA viruses mentioned above, NAT10-mediated ac^4^C modification occurred on the polyadenylated nuclear RNA (PAN RNA) encoded by the oncogenic DNA virus Kaposi’s sarcoma-associated herpesvirus (KSHV) (Yan et al., 2023a). The ac^4^C accumulates at high levels in PAN RNA during viral reactivation, which is crucial for promoting PAN RNA stability, viral gene expression, and virus production. Besides, the upregulating of ac^4^C on tRNA^Ser-CGA-1-1^ also increased the translation efficiency of viral lytic genes and facilitated oncogenic DNA virus KSHV reactivation (Yan et al., 2024).

### DNA

Various chemical modifications naturally occur in genomic DNA (gDNA), exerting significant influence on the biological effects of DNA. Well-studied DNA modifications such as 5-methyldeoxycytosine (^5^mC), 5-hydroxymethyldeoxycytosine (^5^hmC), 5-carboxydeoxycytosine (^5^caC), and N6-methyldeoxyadenosine (^6^mA) have direct analogs in RNA. Recently, a direct analog of ac^4^C called N4-acetyldeoxycytosine (^4^acC) was discovered in *Arabidopsis* DNA (Wang et al., 2022b).

In *Arabidopsis thaliana*, ^4^acC was predominantly found in the euchromatin region and was present in nearly half of the expressed protein-coding genes. ^4^AcC primarily located near the transcription start site, and over half of the ^4^acC peaks co-localize with active histone modification markers possessed, possessing a positive correlation with gene expression levels. However, it is yet to be explored whether ^4^acC modification serves as a universal epigenetic mark associated with gene transcription in other organisms. Further research is needed to elucidate the prevalence and functional significance of ^4^acC modification on DNA across different species and its potential role in gene expression regulation.

### Artificial synthesis

RNA modifications were introduced into mRNA by substituting base-modified nucleoside triphosphates (NTPs) for canonical NTPs using *in vitro* transcription (IVT) reactions generally (Sinclair et al., 2017). It had been utilized to investigate the functional role of ac^4^C modification in synthetic mRNA (Nance et al., 2022). However, traditional methods typically incorporated ac^4^C into RNA in a nonphysiological and uniform manner. Recently, Bartee et al. developed a synthetic pathway for homogeneous RNA containing electrophilic acetyl groups. They employed an orthogonal protection strategy compatible with cytidine acetylation to prevent nucleophilic deprotection that cleaves ac^4^C, allowing for the site-specific synthesis of ac^4^C in RNA (Bartee et al., 2022). This innovative approach enables researchers to explore the impacts of ac^4^C on various functional nucleic acid structures and functions, such as short guide RNA, short interfering RNA, and antisense oligonucleotides in a more precise and controlled manner.

## Identification methods of RNA ac^4^C modification

**Figure 3.**
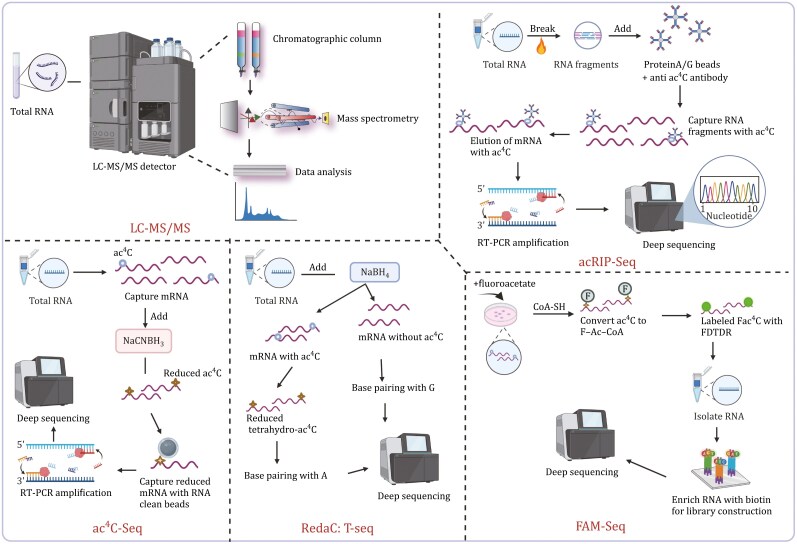
**Detection methods of ac** ^**4**^ **C.** The main procedures of each ac^4^C detection method, categorized based on different principles, are demonstrated (created by BioRender.com).

Early methods for identifying ac^4^C in RNA involved partial enzymatic hydrolysis, two-dimensional thin-layer chromatography, and Deae-cellulose chromatography for benzoylation (Fig. 3). These techniques were used to detect ac^4^C modifications in 5S rRNA and tRNA^ser^ (Bruenger et al., 1993; Kowalski et al., 1971; Stern and Schulman, 1978; Thomas et al., 1978). As liquid chromatography (LC) and mass spectrometry (MS) technologies advanced and the chemical characteristics of ac^4^C were elucidated, along with the development of ac4C-specific antibodies, a variety of new detection techniques have been employed to study ac^4^C (Table 1). The combination of LC, MS, and specific antibodies has greatly expanded the capabilities for detecting and quantifying ac^4^C in RNA, leading to a deeper understanding of its biological significance and functional roles.

**Table 1.** ac^4^C detection methods.

Identification methods	Required sample	ac^4^C detection techniques	Advantages	Disadvantages
Based on chromatography				
UV-LC/UV-HPLC (Sharma et al., 2015; Thomas et al., 2018)	Not mentioned	Separate nucleotides according to the interaction with ultraviolet light 280 nm	1. Large range 2. Localize the specific ac^4^C position	1. Low sensitivity 2. Low efficiency and need extra sample disposing 3. Limited samples
LC-MS/HPLC-MS (Ito et al., 2014a, 2014b; Sharma et al., 2015, 2017)	Total RNA ≥ 2 μg and concentration ≥ 50 ng/μL	Separate nucleotides based on their retention time as they pass through the column and the mass-to-charge ratio of ions	1. Detect compounds with high polarity and poor stability 2. Accurately quantify substances	1. Cannot provide location information 2. Limited sensitivity 3. Complex process
RP-HPLC (Mezzar et al., 2014; Yang et al., 2016)	60 pmol rRNA	Separate nucleotides according to the hydrophilic difference of different compounds on reverse fixation	1. Separate nucleotides fast and convenient 2. Do not rely on expensive MS detectors or radioactive substrates	1. Requires a large amount of solvent for separation 2. Cannot allow the qualitative and quantitative analysis of nucleosides with similar retention times
HPLC-CE (Liebich et al., 2000)	10 mL body fluid	Isolate the analytes by phenylboronate affinity gel chromatography and separation	1. Require low cost with a long service life 2. Need less samples	Using as a complementary technique of RP-HPLC
HPLC-MISPE (Jégourel et al., 2008)	Total RNA: 10 μg	Using a highly specific molecularly imprinted polymer of pyrimidine nucleosides as stationary phase to separate ac^4^C	Need low cost and short time	Cause hydrolysis of ac^4^C
Based on chemical sequencing				
Borohydride-based Sanger sequencing (Thomas et al., 2019)	Total RNA: 3 μg	Chemical reduction of ac^4^C by integrating borohydride with sanger sequencing	1. Single-nucleotide resolution 2. sensitive, require only a few hundred nanograms RNA 3. Quantify modification occupancy at specific residues	1. Require very large read-depth 2. May not pick up lowly expressed RNAs 3. Low efficiency
ac^4^C-Seq (Sas-Chen et al., 2020)	Total RNA > 30 μg mRNA > 5 μg	Chemical reduction of ac^4^C by NaCNBH_3_ integrated with next-generation sequencing	1. Base resolution 2. Detect multiple modifications simultaneously over the entire length of the transcript and avoid the biases introduced by RT and PCR amplification in the conventional RNA-seq workflow	1. Unable to analyze ac^4^C in the densely modified RNAs 2. The number of ac^4^C that has been detected is low
RedaC:T-seq (Beiki et al., 2024; Sturgill et al., 2022)	Cell > 2 × 10^7^ Tissue > 200 mg Total RNA > 10 μg	Selectively reaction of ac^4^C with NaBH_4_, leading to misincorporations during RT, following with next-generation sequencing	Single-base accurate positioning	Mismatched features may not reproducible across samples
RetraC:T-seq (Relier et al., 2024)	Total RNA: 1–10 μg	Improve RedaC:T-seq by improving the mismatch rate of C:T	Single-base accurate positioning	The reliability and robustness need to be confirmed
Based on antibody				
acRIP-Seq (Arango et al., 2018, 2019)	Cell > 2 × 10^7^ Tissue: 500 mg–1 g Total RNA: 30 μg–300 μg mRNA: 10 μg	Enrich RNA fragments by ac^4^C antibody, followed by deep sequencing	1. Fast, straightforward 2. Could generate thousands of ac^4^C-enriched transcribed regions	1. High-input material 2. Limited specificity, the reads may be biased by the affinity of mRNA and the antibody 3. May generate mapping artifacts 4. Lack of stoichiometric information, cannot provide a base-resolution ac^4^C map at the transcriptome level
Based on metabolism				
FAM-Seq (Yan et al., 2023b)	Total RNA: 10 μg	Using fluoroacetyl substrate segments as metabolic markers	Don’t need antibodies, avoid false signals and preference for any sequence	Single-base resolution and labeling efficiency are limited

### Based on chromatography

As a classical method, LC-MS is widely used for detecting ac^4^C in RNA, with applications in rRNA, tRNA, and mRNA across various organisms such as bacteria, yeast, and mammals (Ikeuchi et al., 2008; Ito et al., 2014a, 2014b; Tardu et al., 2019). It involves separating ac^4^C-containing RNA based on retention times in a column, ionizing the molecules, and then analyzing them based on mass-to-charge ratios using a mass spectrometry detector. LC-MS can identify high-polarity chemicals with low stability and accurately measure molecules. The combination of LC-MS/MS with databases like Ariadne allows for unbiased identification and chemical analysis of RNA in complex biological mixtures, which has been employed to ascertain the location of ac^4^C and the acetylase responsible for it on yeast rRNA (Taoka et al., 2014). HPLC-MS/MS (high-performance liquid chromatography coupled with tandem mass spectrometry) is an advanced version of LC-MS/MS that enhances the accuracy of identifying and quantifying RNA modifications (Ito et al., 2014a, 2014b; Sharma et al., 2015, 2017; Su et al., 2014; Taoka et al., 2014). It has been widely used to detect ac^4^C in various RNA molecules and body fluids of mammals and yeast (Sinclair et al., 2017; Thomas et al., 2019; Yang et al., 2016).

Although LC-MS and HPLC-MS are powerful techniques, they have limitations such as complex sample preparation, lack of location information, limited sensitivity, and inability to amplify signals, particularly in detecting ac^4^C modifications on mRNA. To address these limitations, chromatography-based detection methods have been improved by combining them with other technologies. RP-HPLC (reverse-phase high-performance liquid chromatography) is a method that separates compounds based on hydrophobicity differences in the reverse phase, allowing for the sensitive and effective detection of various modified nucleosides in RNA (Mezzar et al., 2014). It had been used to detect ac^4^C on eukaryotic rRNA, including human and yeast (Liebich et al., 2000; Sharma et al., 2015; Yang et al., 2016). HPLC-CE (high-performance liquid chromatography coupled with capillary electrophoresis) is a complementary technique to RP-HPLC that offers advantages such as lower cost and reduced experimental materials and has been used to detect ac^4^C in body fluids like urine from cancer patients. It has been applied to detect the formation of tRNA and 18S rRNA in yeast and humans (Liebich et al., 2000). However, it requires a large amount of solvent for separation and does not allow the qualitative and quantitative analysis of nucleosides with similar retention times (Mezzar et al., 2014; Yang et al., 2016). Another method, HPLC-MISPE (high-performance liquid chromatography coupled with molecularly imprinted solid-phase extraction), developed by Jegourel et al. (2008) is used for detecting ac^4^C in body fluids. While this method is cost-effective and time-efficient, it may lead to the hydrolysis of ac^4^C. These advancements in chromatography-based detection technologies, combined with other techniques, have helped overcome some of the limitations associated with traditional LC-MS and HPLC-MS methods, providing researchers with more options for studying ac^4^C modifications in RNA.

### Based on chemical sequencing

The discovery of a specific reduction reaction between sodium borohydride and ac^4^C in *S*. *cerevisiae* tRNA by Zachau et al. in 1969 laid the foundation for subsequent research (Igo-Kemenes and Zachau, 1969). Thomas et al. later found that reducing ac^4^C with sodium borohydride agents during reverse transcription (RT) led to premature termination and errors in the process (Thomas et al., 2018). Substituting borohydride with NaCNBH_3_ under acidic conditions resulted in faster dynamic changes.

Combining the above chemical signatures with next-generation sequencing techniques gave rise to ac^4^C-seq, a technology capable of transcriptome-wide, single-nucleotide measurement of ac^4^C modifications. Ac^4^C-seq utilizes borohydride to convert ac^4^C to N4-acetyl-3,4,5,6-tetrahydrocytidine, facilitating the determination of ac^4^C locations in RNA through RT (Sas-Chen et al., 2020). Unlike other methods, ac^4^C-seq can detect ac^4^C at the nucleotide level, making it suitable for studying ac^4^C reaction kinetics. Its sensitivity is primarily dependent on stoichiometry and sequencing depth, with potential enhancements through pre-enrichment of samples containing ac^4^C-modified RNA. However, it may underestimate RNA modification levels due to its reliance on a C-to-T detection method (Thalalla Gamage et al., 2021). Moreover, due to the instability of acetamide on ac^4^C and the potential for hydrides to reduce other electron-deficient heteroaromatic rings, the selectivity of the borohydride reduction reaction is limited (Yan et al., 2023b).

Arango et al. developed RedaC:T-seq based on the chemical properties of ac^4^C to map ac^4^C in human mRNA (Arango et al., 2022; Beiki et al., 2024; Sturgill et al., 2022). They employed NaBH_4_ to induce ac^4^C to tetrahydro-ac^4^C, which selectively impacts base pairing during cDNA synthesis. The resulting tetrahydro-ac^4^C pairs with T, while unmodified cytidine continues to pair with G. By integrating Illumina sequencing and comparing the sequencing outcomes of two sample groups, they were able to pinpoint RNA acetylation modification sites in the transcriptome. Additionally, they calculated the extent of RNA acetylation and its influence on RNA expression using RNA-seq data. However, Georgeson and Schwartz challenged this approach, stating that mismatched features were not reproducible across samples, as C>T mismatches were predominantly present in only one of the two biological replicates (Georgeson and Schwartz, 2024). Furthermore, all types of mismatched bases were significantly enriched in wild-type samples, which contradicted the expected acetylation profile. In response, Relier et al. enhanced the RedaC:T method to create a new technique called RetraC:T for ac^4^C detection (Relier et al., 2024). By incorporating an improved dNTP cocktail, they substantially improved the mismatch rate of C:T, achieving stoichiometric detection of ac^4^C in 18S rRNA. Crucially, the utilization of 2-amino-dATP did not lead to cDNA product truncation or an increase in mismatches at other positions. Nevertheless, further evidence is required to validate the accuracy of detecting ac^4^C using the C>T mutation approach in wild-type cells, and additional studies are needed to confirm the reliability and robustness of these detection methods for ac^4^C in RNA.

### Based on antibody

Sinclair et al. leveraged the reducibility of ac^4^C by NaCNBH_3_ to isolate the carrier protein bound with ac^4^C to generate ac^4^C-specific monoclonal antibodies in rabbits (Sinclair et al., 2017). Subsequently, Arango et al. incorporated it with deep sequencing to develop acetylated RNA immunoprecipitation and sequencing (acRIP-seq) technology to enrich ac^4^C sites in human mRNA. The acRIP-seq relies on the specificity of antibodies binding to ac^4^C residues in RNA samples. The process involves extracting RNA from target cells, purifying mRNA via poly(A) selection, fragmenting the RNA, capturing RNA fragments containing ac^4^C modifications with specific antibodies on beads, and then conducting deep sequencing on the isolated captured RNA (Arango et al., 2018, 2019).

The acRIP-seq has been successfully employed to detect ac^4^C in human and viral mRNAs, offering an advantage of signal amplification. However, due to the lower abundance of ac^4^C modifications on eukaryotic mRNA compared to m^6^A, a large initial amount of RNA is required. Additionally, the broad resolution of peaks resulting from next-generation sequencing, typically sequencing RNA fragments at 125 nucleotides, can make it challenging to precisely determine the exact modified residue involved (Arango et al., 2019). Furthermore, the photo-assisted (PA)-ac^4^C-seq method has been utilized to map ac^4^C in HIV-1 mRNA. It involves cross-linking RNAs bound by 4-thiouridine (s4U) labeling and anti-ac^4^C antibodies, followed by RNase footprinting and sequencing to identify ac^4^C sites (Tsai et al., 2020). This approach provides a valuable tool for studying ac^4^C modifications in specific RNA molecules, offering insights into their functional roles in various biological processes.

### Based on metabolism

Yan et al. introduced a method known as FAM-Seq for detecting ac^4^C by utilizing fluoroacetyl substrate segments as metabolic markers (Yan et al., 2023b). In this approach, fluoroacetate is enzymatically converted to its CoA metabolite, fluoroacetyl-CoA (F-Ac-CoA), *in vivo*. The fluoroacetamides generated at ac^4^C sites are subsequently biotinylated via a high-efficiency fluorine-thiol displacement reaction (FDR). By integrating this enrichment process with sequencing, researchers successfully mapped mRNA ac^4^C sites throughout the transcriptome of various human cell lines. One notable advantage of the FAM-Seq method is its antibody-independent nature, eliminating the risk of false signals and sequence bias associated with antibody specificity. However, the technique has limitations in terms of single-base resolution and labeling efficiency, which would benefit from further refinement and optimization to enhance its accuracy and sensitivity in detecting ac^4^C modifications in RNA molecules. This method represents a promising direction in the field of RNA modification detection and could potentially offer valuable insights into the functional roles of ac^4^C in gene expression regulation and other biological processes.

### Computational site prediction

Building upon the work of Arango et al. (2019) regarding ac^4^C on mRNA, Zhao et al. (2019) developed the ac^4^C predictor PACES by combining two random forest classifiers, position-specific dinucleotide sequence profile and K-nucleotide frequencies, to help mining possible ac^4^C motifs on human mRNA. Given the unknown mechanism of ac^4^C synthesis, the predicted ac^4^C sites remain incomplete, with PACES capable of suggesting potential ac^4^C sequences, but not their precise locations. Since PACES predictions are based on only 4,000 human sequences containing ac^4^C in HeLa cells, ac^4^C predictions in other species or cell types should be interpreted with caution.

In recent years, several novel computational models have been developed to effectively detect ac^4^C sites in human mRNA using machine learning techniques. These models include (Table 2):

**Table 2.** Computational prediction methods of ac^4^C.

Methods	Principles to identify ac^4^C
PACES (Zhao et al., 2019)	The k-nucleotide frequency and the known ac^4^C characteristics
DeepAc4C (Wang et al., 2022a)	Physicochemical patterns and distributed characterization information
Stacking-ac4C (Lou et al., 2023)	Kmer, electron–ion interaction pseudo-potential values (PseEIIP), pseudo-K tuple nucleotide composition (PseKNC), robust Cluster Centroids algorithm combination
iRNA-ac4C (Su et al., 2023)	Nucleotide composition, nucleotide chemical property, and accumulated nucleotide frequency
LSA-ac4C (Lai and Gao, 2023)	Double-layer Long Short-Term Memory (LSTM) and self-attention mechanism
TransAC4C (Liu et al., 2024a)	Transformer-based architecture and pipeline
MetaAc4C (Li et al., 2024)	BLSTM deep learning model that leverages pre-trained bidirectional encoder representations from transformers (BERT)
ac4C-AFL (Pham et al., 2024)	16 feature descriptors with a unique EFIS and AB algorithm
Voting-ac4C (Jia et al., 2024)	RNAErnie pre-trained transformer and six traditional feature extraction methods (such as One-hot, ENAC, etc.) combination
RMDisease V2.0 (Song et al., 2023)	A database with deep learning model

1) DeepAc4C: A convolutional neural network model that identifies ac^4^C in mRNA based on physicochemical patterns and distributed characterization information (Wang et al., 2022a).
2) Stacking-ac4C: A model integrated Kmer, electron–ion interaction pseudo-potential values (PseEIIP), pseudo-K tuple nucleotide composition (PseKNC) to identify ac^4^C in human mRNA, and combined with robust Cluster Centroids algorithm to improve imbalanced data processing (Lou et al., 2023).
3) iRNA-ac4C: A model that identifies ac^4^C sites in human mRNA using three feature extraction methods—nucleotide composition, nucleotide chemistry, and cumulative nucleotide frequency (Su et al., 2023).
4) LSA-ac4C: A model that identifies ac^4^C sites in human mRNA by combining double-layer Long Short-Term Memory (LSTM) and self-attention mechanism (Lai and Gao, 2023).

Furthermore, there are other computational models continuously being improved, such as TransAC4C (Liu et al., 2024a), MetaAc4C (Li et al., 2024), and ac^4^C-AFL (Pham et al., 2024), and Voting-ac4C (Jia et al., 2024), each offering unique approaches to predicting ac^4^C sites within mRNA. In addition, the RMDisease V2.0 database has been developed to explore functional associations between RNA modifications (RMs) and various human diseases, shedding light on the link between ac^4^C modifications and genetic variations underlying the pathogenesis of human diseases (Song et al., 2023).

## Roles of RNA ac^4^C modification in reproductive health

Disturbances in the reproductive process and gynecological diseases are significant factors impacting reproductive health. Numerous studies have emphasized the critical roles of mRNA modifications in human fertility. The extensively studied m^6^A modification has been demonstrated to play essential regulatory roles in spermatogenesis, oogenesis, embryo development, and cell fate transitions. This highlights the involvement of posttranscriptional epigenetic regulation in reproductive health (Batista et al., 2014; Geula et al., 2015; Xu et al., 2017; Tang et al., 2018). As ac^4^C garners increasing attention as a novel mRNA modification, its association with reproductive health has also been elucidated. The exploration of ac^4^C’s role in reproductive processes may provide further insights into the intricate mechanisms governing fertility and reproductive health.

### Roles of RNA ac^4^C modification in reproductive process

**Figure 4.**
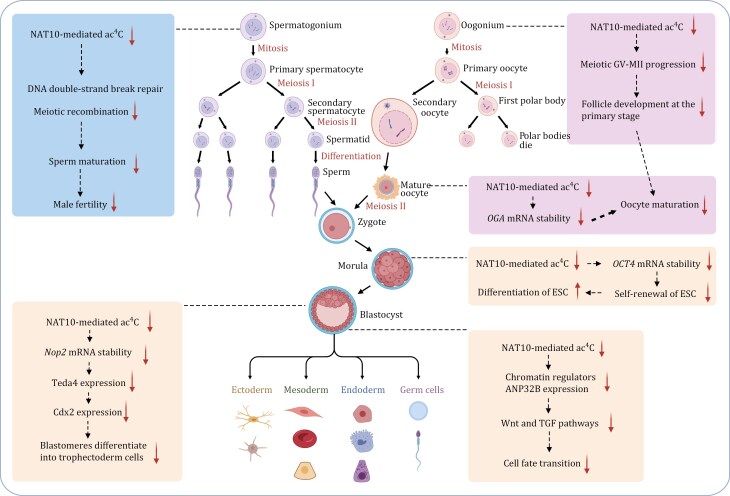
**Roles of ac** ^**4**^ **C in reproductive process.** The functions of ac^4^C in reproductive development, including the entire process from the formation of ovum and sperm to the differentiation of embryos, are depicted. Each function at various stages is represented in different boxes (created by BioRender.com).

Germ cell lineages undergo oogenesis and spermatogenesis to produce eggs and sperm, which unite during fertilization to form an embryo (Fig. 4). Following implantation, the embryo undergoes development and differentiation into three germ layers. Disruptions at any of these stages can lead to reproductive issues such as infertility or birth defects. The ac^4^C modification plays crucial roles in various stages of this intricate reproductive process.

#### In oogenesis

During oogenesis, the development of mature oocytes from primordial germ cells through meiosis is a crucial process in reproductive health. Recent research by Xiang et al. has highlighted the involvement of ac^4^C modification and NAT10 expression in oocyte maturation (Xiang et al., 2021). They demonstrated a decrease in both ac^4^C and NAT10 levels as mouse oocytes matured. A reduction in ac^4^C levels and delayed meiotic maturation *in vitro* were observed when NAT10 was knocked down by small interfering RNAs (siRNAs) in GV stage oocytes, emphasizing the critical regulatory role of NAT10-mediated ac^4^C modification during oocyte maturation. Furthermore, Xiang et al. utilized RNA pull-down technique and bioinformatics analyses in HEK293T cells to identify genes modulated by ac^4^C that are associated with nucleosome assembly, chromatin silencing, and chromatin modification. They proposed Transducin beta-like protein 3 (TBL3) as a potential ac^4^C-binding protein during oocyte maturation, although direct confirmation of TBL3 binding to ac^4^C and its regulatory role was not determined.

Additionally, Lin et al. identified *O-GlcNAcase* (*OGA*) as a key target gene for NAT10-mediated ac^4^C (Lin et al., 2022). They observed an increase in *OGA* expression during oocyte maturation, and knockdown of *OGA* hindered oocyte maturation, underscoring the importance of *OGA* in this process. NAT10-mediated ac^4^C appears to play a role in maintaining the stability of the *OGA* transcript, thereby promoting oocyte maturation. Furthermore, *Trpc7* and *Rsph6a* were identified as potential downstream genes in this pathway. Genetic evidence further supports the essential role of NAT10 in oocyte growth and maturation (Jiang et al., 2023). Loss of NAT10 before meiosis resulted in the cessation of follicle development at the primary stage and premature ovarian failure (POF), and *in vitro* ablation of NAT10 in GV oocytes impaired meiotic progression from GV to MII stage. Collectively, these findings highlight the significance of NAT10-mediated ac^4^C in the progression of meiosis prophase I in the female embryonic gonad. Understanding the function of ac^4^C in regulating oocyte maturation not only provides insights into fundamental reproductive processes but also offers potential applications in improving *in vitro* oocyte maturation (IVM) and enhancing artificial fertilization techniques.

#### In spermatogenesis

Spermatogenesis is a highly orchestrated process involving the differentiation of diploid spermatogonium stem cells (SSC) into various stages of spermatogonia, culminating in the production of mature sperm through meiosis. Any disruptions in this intricate process can lead to male infertility. Chen et al. have highlighted the significance of ac^4^C expression in the epididymis and testes, with dynamic changes observed in its levels during spermatogenesis (Chen et al., 2022). Their study demonstrated that male mice lacking NAT10 exhibited notably smaller testes and a lack of mature sperm in the epididymis, resulting in complete male infertility. Moreover, when NAT10 was knocked out during meiosis, abnormalities were observed in homologous chromosomal synapsis, meiotic recombination, and DNA double-strand break repair. Additionally, a decrease in the expression of key proteins essential for meiosis was determined. These findings suggest that the deletion of NAT10 impacts spermatogonial differentiation and meiotic entry, emphasizing the crucial role of NAT10-mediated ac^4^C in spermatogenesis and male fertility. Understanding the mechanisms underlying ac^4^C modification may offer valuable insights into potential therapeutic avenues for addressing male infertility stemming from disruptions in spermatogenesis.

#### In early embryo development

During early embryonic development, the maintenance of self-renewal and pluripotency in embryonic stem cells, particularly at the morula and blastocyst stages, is crucial for proper embryo formation. Liu et al. established the NAT10-knockdown human embryonic stem cells (hESC) and revealed that the loss of NAT10 function led to a depletion of self-renewal capacity and pluripotency in hESC (Liu et al., 2023). Notably, the downregulation of NAT10 resulted in a significant reduction in the expression level and mRNA stability of *OCT4*, a key regulator of pluripotency. This highlighted the role of NAT10-mediated ac^4^C modification in regulating hESC self-renewal by preserving the mRNA stability of the essential pluripotency factor OCT4. Moreover, based on the analysis of the ac^4^C landscape in early mouse embryos, Wang et al. found embryos deficient in NAT10 exhibited a failure to progress into normal blastocysts (Wang et al., 2023b). The study further revealed that disrupted ac^4^C modification of *Nop2* mRNA impeded the transition from the morula to blastocyst stage in mice, impacting the initial cell fate determination process. The mechanism elucidated by the researchers indicated that NOP2 depletion hindered the translation of the transcription factor TEAD4, leading to compromised expression of the downstream lineage-specific gene *Cdx2*. This disruption prevented blastomeres from differentiating into trophectoderm, thereby impeding the formation of blastocysts. The study underscored the necessity of *Nop2* mRNA ac^4^C for the morula-to-blastocyst transition, emphasizing the significance of ac^4^C modification in mammalian preimplantation embryogenesis. Hu et al. (2024) also clarified that NAT10-mediated ac^4^C controls hESC cell fate via regulating chromatin signaling. They observed the strongly enriched of ac^4^C for fate-instructive chromatin regulators and verified the histone chaperone ANP32B to be the key downstream targets of NAT10. In particular, the NAT10–ac^4^C–ANP32B axis regulates the chromatin landscape of downstream genes to regulate key pathways including Wnt and TGF pathways during cell fate transitions.

### Roles of RNA ac^4^C modification in gynecological diseases

**Figure 5.**
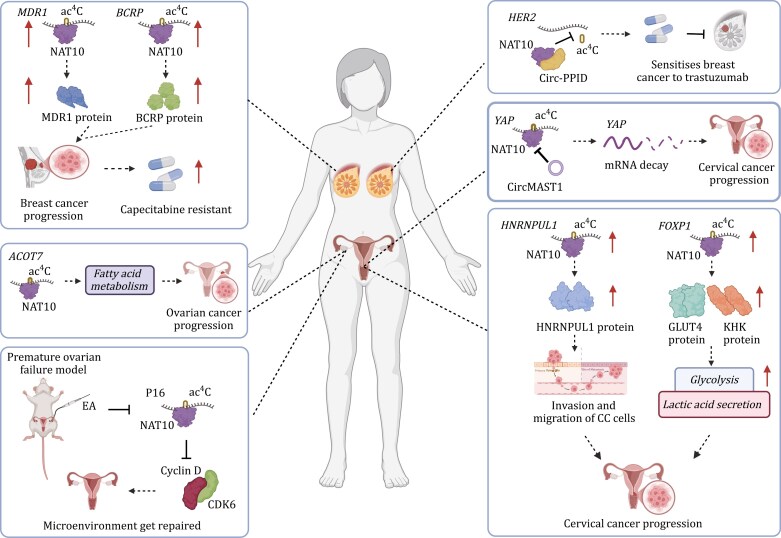
**Roles of ac** ^**4**^ **C in gynecological diseases.** The functions of ac^4^C in gynecological diseases, including breast diseases, ovarian disease, and uterus diseases, are shown, respectively (created by BioRender.com).

Gynecological diseases including vulvar diseases, vaginal diseases, uterine diseases, fallopian tube diseases, ovarian diseases, etc., are posing serious threats to women’s health (Fig. 5). NAT10-mediated ac^4^C has been proved to be involved in the genesis and development of several gynecological diseases through diverse biological mechanisms.

#### In cervical cancer

Cervical cancer (CCa) stands as the most prevalent malignant neoplasm affecting the female reproductive tract, characterized by the highest rates of morbidity and mortality among women. Notably, elevated NAT10 expression in CCa tissues has been linked clinically to an unfavorable prognosis (Chen et al., 2023). Chen et al. elucidated that this phenomenon stems from the activation of NAT10 through its binding to the promoter region with the transcription factor HOXC8. Consequently, NAT10 catalyzes the ac^4^C modification of *FOXP1* mRNA, enhancing its translational efficiency and subsequently upregulating *GLUT4* and *KHK* expression, thereby driving CCa progression. Moreover, the NAT10–ac^4^C–FOXP1 axis has been identified to augment lactic acid production and bolster glycolytic activity in CCa cells, thereby intensifying the immunosuppressive characteristics of tumor-infiltrating regulatory T cells.

Additionally, Long et al. unveiled the targeting of HNRNPUL1 by NAT10 in CCa (Long et al., 2023). The promotion of CCa development by NAT10 occurs through the enhancement of HNRNPUL1 mRNA stability via ac^4^C modification. The NAT10–ac^4^C–HNRNPUL1 axis emerges as a promising therapeutic target for CCa treatment. Furthermore, Zhang et al. revealed that CircMAST1 plays a role in reducing tumor progression and lymph node metastasis in CCa (Zhang et al., 2024a). The circMAST1 selectively binds to NAT10, inhibiting the ac^4^C modification of Yes-associated protein (YAP) mRNA, thereby promoting its degradation and impeding tumor development in CCa.

Collectively, these findings underscore the oncogenic implications of NAT10-mediated ac^4^C modifications in CCa progression, its interplay with immunosuppression, and suggest its potential as a synergistic target for PD-1/PD-L1 blockade immunotherapy in CCa.

#### In POF and ovarian cancer

POF poses a significant challenge to women’s fertility, and electroacupuncture (EA) has emerged as a potential therapeutic intervention for this condition. Geng et al. proposed a mechanism wherein EA may alleviate POF through ac^4^C modification (Geng et al., 2022). Their research indicated that EA facilitated the restoration of the ovarian microenvironment by suppressing the ac^4^C modification of *P16* mRNA, leading to reduced stability and expression levels, consequently resulting in elevated expression of Cyclin D (CCND1) and CDK6. The downregulation of NAT10 was found to modulate the P16–CDK6–CCND1 axis activity in ovarian granulosa cells (OGCs), thereby aiding in the restoration of the ovarian microenvironment.

Ac^4^C level displayed noticeable alterations in urine and blood of patients with ovarian cancer (OC). Szymańska et al. (2010) collected urine of patients with urogenital tract cancer for composing urine profiles of urinary nucleotides sides and found that the ac^4^C content was significantly increased. Zhang et al. (2012) observed the similar increase of ac^4^C level in the urine of patients with epithelial OC. In a related study, Zheng et al. uncovered a link between ac^4^C modification and OC by employing the non-negative matrix factorization (NMF) method to analyze RNA modifications in OC samples (Zheng et al., 2022). In terms of mechanism, Liu et al. (2024c) reported that NAT10 increases the *ACOT7* mRNA stability via mediating ac^4^C modification to suppress ferroptosis and modulate fatty acid metabolism in OC cells, thereby promoting tumorigenesis. They also identified fludarabine as a small molecule inhibitor targeting NAT10 to effectively suppress ovarian tumorigenesis.

#### In breast cancer

In breast cancer, elevated NAT10 expression has been observed across all breast cancer cell lines, and inhibiting NAT10 expression has shown to effectively suppress breast cancer cell proliferation and invasion, as reported by Zhao et al. (2024). The study further unveiled that NAT10 acetylates the mRNA of multidrug resistance protein 1 (MDR1) and breast cancer resistance protein (BCRP), leading to increased expression levels that drive breast cancer progression. Moreover, the use of NAT10 inhibitors has demonstrated the restoration of sensitivity in capecitabine-resistant breast cancer cells to chemotherapy, both *in vitro* and *in vivo*. Resistance to trastuzumab in human epidermal growth factor receptor 2 (HER2) positive breast cancer is a significant clinical challenge, often associated with HER2 overexpression and activation. Wang et al. (2024) shed light on a novel mechanism involving circ-PPID (peptidylprolyl isomerase D circular RNA) in sensitizing breast cancer cells to trastuzumab treatment by modulating HER2 ac^4^C modification. Circ-PPID was found to reduce HER2 mRNA ac^4^C levels by binding to NAT10, thereby enhancing the efficacy of trastuzab. These findings provide valuable insights into the role of ac^4^C modifications in breast cancer development and drug resistance.

## Conclusions and prospects

In this review, we have recapitulated the current research progress on ac^4^C modification in RNA and its role in regulating gametogenesis, embryo development, and gynecological diseases in eukaryotes. This exploration not only enhances our comprehension of the biological processes modulated by ac^4^C but also advances our understanding of embryo differentiation and development. It offers new research perspectives for the field of reproductive medicine and the diagnosis and treatment of gynecological diseases. However, research in this field is still at a nascent stage, and the application of ac^4^C in the etiological diagnosis and gene therapy of infertility diseases faces several challenges that need further elucidation. Moreover, NAT10 is currently the sole known ac^4^C writer enzyme in mammals, while the eraser and reader for ac^4^C, along with other potential cofactors of NAT10, remain unknown. Delving into the detailed mechanisms underlying ac^4^C addition, removal, and recognition presents an intriguing avenue for future investigations. Furthermore, although ac^4^C has been confirmed to exist in miRNA and lncRNA in mammals and reported as ^4^acC in *Arabidopsis thaliana* DNA, its presence and distribution in other RNA types and species, as well as its precise biological function in epigenetic regulation, necessitate further independent research for clarification.

Additionally, numerous studies underscore the pivotal role of other mRNA modifications, particularly m^6^A, in human fertility. It is evident that m^6^A and ac^4^C share several similar functions in the reproductive process, such as regulating mRNA stability, ESC pluripotency and differentiation, as well as oogenesis and spermatogenesis. There is a possibility that these two RNA modifications may exhibit a synergistic effect or mutual inhibition. However, the interplay between ac^4^C modification and other RNA modifications requires further investigation. Further exploration of the relationship between ac^4^C and other base modifications may unveil the mechanisms underlying ac^4^C function in biological processes and offer fresh insights for the prevention and treatment of reproductive diseases.

Future research in the field of epitranscriptomics will likely rely on advancements in single-cell and single-molecule approaches, as well as the development of novel sequencing technologies capable of detecting multiple modifications simultaneously. This approach will enhance our understanding of potential cooperative or exclusive relationships between various modified nucleotides. Third-generation sequencing technologies, such as Single-Molecule Real-Time (SMRT) sequencing and Nanopore technology, hold promise for revolutionizing epitranscriptomics research. However, challenges such as complex algorithms, low signal-to-noise ratios, high error rates, and high costs currently limit their application in ac^4^C detection. Overcoming these obstacles will be crucial for harnessing the full potential of these technologies in studying RNA modifications. Additionally, spatial epitranscriptome profiling is expected to emerge as a significant area of study for understanding mRNA modification profiles at the level of individual cells in both spatial and temporal dimensions. This approach will provide valuable insights into how mRNA modifications vary across different cell types and developmental stages.

In conclusion, the ongoing development of epitranscriptomic techniques is a critical pathway for advancing our understanding of mRNA alterations and their roles in gene regulation, cellular processes, and disease mechanisms. Continued innovation in technology and methodology will be essential for unlocking the full potential of epitranscriptomics in unraveling the complexities of RNA modifications.

## Data Availability

The data are all available in the article.
